# Safety of live attenuated influenza vaccine in young people with egg allergy: multicentre prospective cohort study

**DOI:** 10.1136/bmj.h6291

**Published:** 2015-12-08

**Authors:** Paul J Turner, Jo Southern, Nick J Andrews, Elizabeth Miller, Michel Erlewyn-Lajeunesse

**Affiliations:** 1 Section of Paediatrics, Imperial College London, London W2 1PG, UK; 2 Immunisation Hepatitis and Blood Safety Department, Public Health England, London, UK; 3 Paediatric Allergy and Immunology, Southampton University Hospital, Southampton, UK

## Abstract

**Study question** How safe is live attenuated influenza vaccine (LAIV), which contains egg protein, in young people with egg allergy?

**Methods** In this open label, phase IV intervention study, 779 young people (2-18 years) with egg allergy were recruited from 30 UK allergy centres and immunised with LAIV. The cohort included 270 (34.7%) young people with previous anaphylaxis to egg, of whom 157 (20.1%) had experienced respiratory and/or cardiovascular symptoms. 445 (57.1%) had doctor diagnosed asthma or recurrent wheeze. Participants were observed for at least 30 minutes after vaccination and followed-up by telephone 72 hours later. Participants with a history of recurrent wheeze or asthma underwent further follow-up four weeks later. The main outcome measure was incidence of an adverse event within two hours of vaccination in young people with egg allergy.

**Study answer and limitations** No systemic allergic reactions occurred (upper 95% confidence interval for population 0.47% and in participants with anaphylaxis to egg 1.36%). Nine participants (1.2%, 95% CI 0.5% to 2.2%) experienced mild symptoms, potentially consistent with a local, IgE mediated allergic reaction. Delayed events potentially related to the vaccine were reported in 221 participants. 62 participants (8.1%, 95% CI for population 6.3% to 10.3%) experienced lower respiratory tract symptoms within 72 hours, including 29 with parent reported wheeze. No participants were admitted to hospital. No increase in lower respiratory tract symptoms occurred in the four weeks after vaccination (assessed with asthma control test). The study cohort may represent young people with more severe allergy requiring specialist input, since they were recruited from secondary and tertiary allergy centres.

**What this study adds** LAIV is associated with a low risk of systemic allergic reactions in young people with egg allergy. The vaccine seems to be well tolerated in those with well controlled asthma or recurrent wheeze.

**Funding, competing interests, data sharing** This report is independent research commissioned and funded by a Department of Health policy research programme grant to the National Vaccine Evaluation Consortium. Additional funding was provided by the NIHR Clinical Research Networks, Health Protection Scotland (Edinburgh site), and Health & Social Care Services in Northern Ireland (Belfast site). PJT and MEL had support from the Department of Health for the submitted work; PJT has received research grants from the Medical Research Council and NIHR. No additional data available.

**Study registration** ClinicalTrials.gov (NCT02111512) and the EU Clinical Trials Register EudraCT (2014-001537-92).

## Introduction

Epidemiological data and mathematical modelling indicate children are the main spreaders of influenza infection.^1^ Vaccinating children therefore provides the most effective method for interrupting transmission and achieving disease control. This was recognised by the Joint Committee for Vaccination and Immunisation, an independent expert advisory committee to the UK Departments of Health, which in 2012 recommended annual vaccination of all children aged 2-16 years with the live attenuated influenza vaccine (LAIV).^2^ This vaccine is given through the intranasal route and has high efficacy against influenza in young people aged 2-17 years,^3^ ^4^ with a good safety profile, similar to that of inactivated influenza vaccines.^5^ ^6^ ^7^ ^8^ ^9^ The Joint Committee for Vaccination and Immunisation considered that extending the influenza vaccine programme to include both high risk and low risk children was likely to be safe and cost effective, providing direct protection to the vaccinated child as well as indirect protection by lowering influenza transmission from vaccinated children and adolescents to others, including those in clinical risk groups.^2^

In common with other influenza vaccines licensed for use in young people, LAIV is grown in hens’ eggs and contains egg proteins, such as ovalbumin.^10^ There is now a consensus that inactivated influenza vaccines with a low ovalbumin content (<0.12 μg/mL, equivalent to 0.06 μg for a 0.5 mL dose) are safe for use in people with egg allergy,^11^ ^12^ with the proviso that “in all settings providing vaccination, facilities should be available and staff trained to recognise and treat anaphylaxis.”^11^ Until recently, there was no safety data on the use of LAIV in young people with egg allergy, and egg allergy remains listed as a contraindication for LAIV in the summary of product characteristics.^10^ For the 2015/16 influenza season, seasonal influenza vaccination will be offered to all 2 to 4 year olds, and those in school years 1 and 2, using quadrivalent LAIV unless otherwise contraindicated.^13^ The prevalence of egg allergy is estimated to be 2.5% in this age group,^14^ so on the basis of UK 2013 population data, there are 100 000 young people with egg allergy in whom vaccination with LAIV would therefore be contraindicated.

Young people with egg allergy often have concomitant diseases, including eczema and recurrent wheeze. Some guidelines recommend against the use of LAIV in young people with recurrent wheeze, owing to limited evidence from a clinical trial^15^ that the vaccine may induce wheezing in younger children.^16^ These are important barriers to achieving successful implementation of the immunisation programme in the community and primary care settings. To address this and provide data to underpin an evidence based change in guidance, we assessed the safety of using LAIV in young people with egg allergy in a large, multicentre, interventional study.

## Methods

We undertook a phase IV open label study of LAIV in young people with egg allergy, during the influenza season (September 2014 to February 2015) across 30 hospitals (specialist and non-specialist clinics) in the United Kingdom. Eligible participants were aged 2-18 years, with a current doctor diagnosis of egg allergy. We also included young people with a history of anaphylaxis to egg or a history of severe but stable asthma. Anaphylaxis was defined using World Allergy Organization criteria.^17^ We classified asthma according to current treatment at time of immunisation, using the British Thoracic Society (BTS) and Scottish Intercollegiate Guidelines Network (SIGN) guidelines.^18^

Participants were excluded if they had previously required invasive ventilation for an anaphylactic reaction to egg, had severe asthma (defined as BTS/SIGN step 5 treatment with poor control, assessed by the attending specialist or with an asthma control test score of <20),^19^ or contraindication to LAIV (other than egg allergy). Vaccination was deferred for acute febrile illness; wheeze in the preceding 72 hours, or acute asthma symptoms requiring corticosteroids in the previous two weeks; and receipt of antihistamine within the previous four days (owing to the possibility that any allergic symptoms might be masked).

The parent or guardian of each participant gave written informed consent. Young people over 8 years were encouraged to provide assent. The study was registered with ClinicalTrials.gov (NCT02111512) and the EU Clinical Trials Register EudraCT (2014-001537-92).

### Procedures

We recorded baseline measurements (blood pressure, heart rate, respiratory rate, oxygen saturations), with simultaneous clinical respiratory and dermatological assessment. Quadrivalent LAIV (Fluenz Tetra, MedImmune LLC, Nijmegen, Netherlands; distributed by Astra Zeneca, London UK; produced for the 2014/15 influenza season) was administered according to the approved summary of product characteristics.^10^ We observed participants for at least 30 minutes for symptoms of local or systemic allergic reaction, and documented clinical observations and symptom scoring (total nasal symptom score)^20^ on a dedicated study case report form. After at least 72 hours we telephoned the parents to document any delayed symptoms. In participants with a history of asthma or recurrent wheeze, we administered the asthma control test before vaccination and four weeks later. This test is a validated tool providing an assessment of asthma symptoms over the preceding four weeks.^19^ We offered a second dose of LAIV to participants in a high risk clinical group who had not received a previous seasonal influenza vaccine at least four weeks later, in line with national guidelines.^12^

### Outcomes

The primary outcome was the incidence of allergic reaction as an adverse event following immunisation (AEFI) occurring within two hours of vaccination, according to symptoms listed in the Brighton Collaboration case definition for systemic allergic reaction (anaphylaxis) as an AEFI.^21^ We defined any reaction not meeting the case definition for anaphylaxis as a possible non-anaphylactic reaction. A change in total nasal symptom score of 3 or more was taken as indicative of a possible local (nasal) allergic response.^20^

Secondary outcomes were incidence of delayed symptoms occurring up to 72 hours after vaccination with LAIV (including those related to non-allergic factors); change in asthma control test score before and one month after vaccination in participants with a history of asthma or recurrent wheeze, or both. In children under 12 years, we compared only the subscore relating to parental assessment of symptoms. An independent data monitoring committee reviewed all adverse events, and assigned causality in conjunction with local study teams.

### Statistical analyses

Analyses were planned prospectively and detailed in a statistical analysis plan. We estimated the incidence of reactions to LAIV (both immediate and delayed) with two sided exact 95% confidence intervals. For subgroup analyses, we used a two sided Fisher’s exact test to compare the incidence of reactions between different cohorts. Subgroup analyses included age group (2­5, 6­11, 12­17 years), certainty of true clinical allergy (on the basis of reaction to egg within the previous 12 months and/or evidence of >95% likelihood of egg allergy according to published criteria),^22^ ^23^ history of anaphylaxis to egg, history of previous reaction to airborne traces of egg, tolerance to extensively heated egg, previous receipt of any influenza vaccine (inactivated or LAIV) or LAIV alone, presence of doctor diagnosed asthma or recurrent wheeze, and ovalbumin content of LAIV batch used. We used McNemar’s exact test to assess change in asthma control test score.

Sample size was considered for historical comparisons and was based on the precision around an estimate of zero. If there were no allergic reactions in a sample size of 730, then this would provide confidence (based on the upper end of the two sided 95% confidence interval) that the true rate of allergic reaction to LAIV in young people with egg allergy within the population was no more than 0.5%. The analysis dataset was as treated and with the relevant safety data measured.

### Patient involvement

Parents of young people with egg allergy were involved in the design of the study and the development of study information leaflets, and in setting the research question. Results of the study will be disseminated through patient support organisations (Allergy UK and the Anaphylaxis Campaign) through electronic newsletters and social media.

## Results

Overall, 779 children with egg allergy were enrolled and received at least one dose of LAIV between September 2014 and February 2015. The median age of the cohort was 5.3 years (range 2-18 years) and 508 (65.2%) were male. Three hundred and sixty nine (47.4%) had received influenza vaccination in previous years, of whom 188 had been given LAIV. Most LAIV in circulation in the UK does not contain detectable ovalbumin (personal communication, Department of Health). For this study, we sourced vaccine with detectable ovalbumin. In 667 (85.6%) children the batch of LAIV used contained >0.3 ng/mL ovalbumin, and 511 (65.6%) received a dose containing 1-1.93 ng/mL ovalbumin.

All the participants were excluding egg from their diet at the time of immunisation. Three hundred and fifteen (40.4%) had experienced an allergic reaction to egg in the past 12 months, including 40 (5.1%) at formal, in-hospital food challenge. A total of 138 (17.7%) had not reacted to egg in the past 12 months but had evidence of sensitisation above the published criteria for more than 95% positive predictive values for clinical egg allergy.^22^ ^23^ Thus, 453 (58.2%) young people met the criteria consistent with more than a 95% likelihood of clinical egg allergy within the 12 months before vaccination. An allergy specialist verified the egg allergy diagnosis in 673 (86.4%) participants. Of the remainder, 90 had received a diagnosis by a general paediatrician and 16 by their general practitioner; within this subgroup, only 42 (5.3% of total cohort) did not meet the 95% positive predictive value criteria.

The cohort included 270 (34.7%) young people with a history of anaphylaxis to egg, of whom 157 (20.1%) had experienced respiratory and/or cardiovascular symptoms with egg ingestion. Fifty three (6.8%) participants had experienced World Allergy Organization grade 3+ reactions (stridor with respiratory compromise, wheeze not responsive to initial bronchodilator treatment, or collapse/hypotension). Only 56 (7.2%) had never eaten egg and had a diagnosis based on predictive allergy testing alone. Four hundred and forty five participants (57.1%) had a doctor diagnosis of asthma or recurrent wheeze, of whom 361 (46.3% of total cohort) were using daily preventer treatment (BTS/SIGN step 2+) and 143 (18.4%) BTS/SIGN step 3+ treatment. Three hundred and seventy seven (48.4%) had allergic rhinitis, 463 (59.4%) had atopic eczema, and 435 (55.8%) were allergic to three or more food groups.

A second LAIV dose was administered to 30 young people: 28 vaccine naïve participants who required a further dose according to clinical risk, and two participants who underwent subsequent allergy skin testing, including nasal challenge with vaccine, owing to possible systemic allergic reaction to LAIV (fig 1). A further 15 children were eligible for a second dose, but did not receive it owing to expiry of the vaccine (nine participants) or the family declining a second visit for a further dose (six participants).

**Figure fig1:**
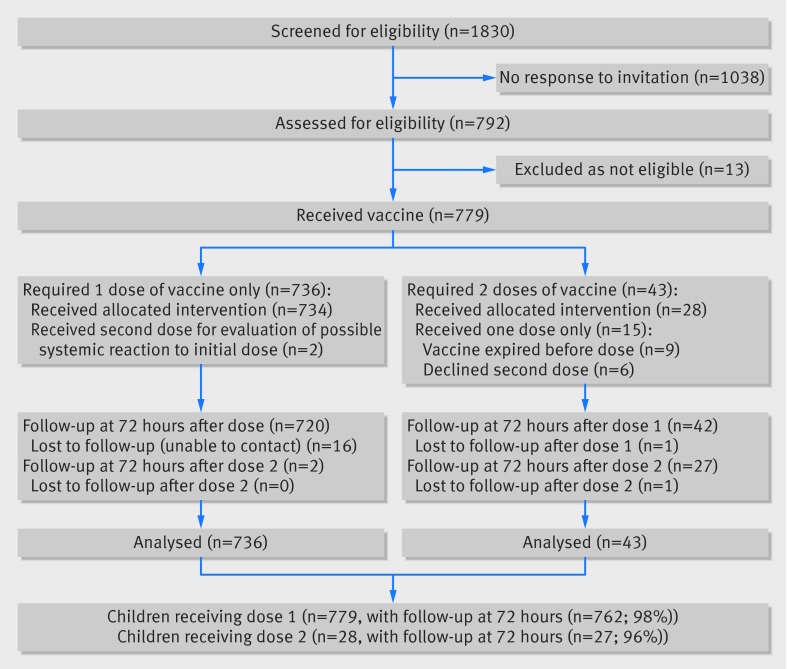
**Fig 1** Flow of participants through study. 13 children were consented but not eligible for study: six had recently used antihistamines (family declined further study appointment), five refused after parental consent had been obtained; two had unstable asthma (and were given inactivated influenza vaccine instead)

### Primary outcome: immediate adverse events following immunisation

Seventeen adverse events were recorded in 17 individual participants within two hours of vaccination (see supplementary table E1). Six were not consistent with a potential, IgE mediated allergic response as defined by international consensus criteria.^21^ Two participants reported skin symptoms (urticaria/angioedema) between 30 and 120 minutes after LAIV; both underwent subsequent specialist allergy testing four weeks later (to LAIV and excipients, all of which produced negative results) and were given a second dose of LAIV which was tolerated without any observed adverse symptoms in the two hours after vaccination. In one case, the initial reaction could be attributed to unintentional consumption of cow’s milk, to which the participant was allergic. Therefore, no participant experienced a systemic reaction attributed to LAIV; the 95% upper confidence interval for the incidence of a systemic allergic reaction (including anaphylaxis) to LAIV in participants with egg allergy was therefore 0.47%. In participants with a history of anaphylaxis, the equivalent 95% upper confidence interval was 1.36%.

Nine participants (1.2%, 95% confidence interval 0.5% to 2.2%) experienced an immediate AEFI of possible allergic cause. These reactions (four rhinitis, four localised/contact urticaria, one oropharyngeal itch) were mild, self limiting, and occurred within 30 minutes of immunisation with LAIV. Participants with a history of reaction to aerosolised egg had a higher incidence of possible reaction (3/70 *v* 6/709, P=0.04), but otherwise no risk factors were identified for occurrence of an acute adverse event, allergic or otherwise, when participants were assessed for age, previous anaphylaxis to egg, previous influenza vaccination (any, or previous LAIV), presence of doctor diagnosed asthma or recurrent wheeze or allergic rhinitis, or level of ovalbumin in the administered dose of LAIV (P>0.05 for all comparisons, see supplementary table E2).

### Delayed adverse events (2-72 hours after vaccination)

No serious adverse events attributable to LAIV occurred during the study. Delayed events potentially related to the vaccine were reported in 221 participants (table 1). Sixty two children (8.1%, 95% confidence interval for population 6.3% to 10.3%) experienced lower respiratory tract symptoms within 72 hours after vaccination, including 29 with parent reported wheeze (3.8%, 2.6% to 5.4%). No risk factors were identified for occurrence of delayed events, although there was a trend towards an increased rate of lower respiratory tract symptoms in younger children (P=0.07, see supplementary table E3). Some guidelines have suggested that children under 5 years with a history of wheezing are at risk of developing wheeze after vaccination with LAIV. To assess this, in an additional exploratory analysis we compared the rate of lower respiratory tract symptoms in children with asthma or recurrent wheeze: children under 5 years were slightly more likely to develop lower respiratory tract symptoms compared with older children, although this did not reach significance (22/149 (15%) children under 5 years versus 26/296 (8.7%), P=0.07). Parents sought medical review by the child’s primary care doctor in five cases, resulting in a change in management in three; one child was referred to hospital for further assessment but was discharged without admission after review.

**Table 1 tbl1:** Delayed adverse events 2-72 hours after immunisation with LAIV as reported by parents

Adverse events	No of children (n=762)	% rate (95% CI) in cohort
Upper respiratory tract:	****	****
Any symptoms	141	18.5 (15.8 to 21.4)
Isolated symptoms only, <24 hours duration	72	9.4 (7.5 to 11.8)
Isolated symptoms only, >24 hours duration	69	9.1 (7.1 to 11.3)
Nasal symptoms with ocular involvement	1	0.1 (0.0 to 0.7)
Lower respiratory tract:	****	****
Any symptoms	62	8.1 (6.3 to 10.3)
Parent reported wheeze	29	3.8 (2.6 to 5.4)
Constitutional symptoms:	****	****
Any	53	7.0 (5.2 to 9.0)
Fever <24 hours	30	3.9 (2.7 to 5.6)
Fever >24 hours	9	1.2 (0.5 to 2.2)
Other: lethargy, headache, dizziness, myalgia	19	2.5 (1.5 to 3.9)
Dermatological:	****	****
Flare in eczema	22	2.9 (1.8 to 4.3)
Non-specific rash, no response to antihistamine	8	1.0 (0.5 to 2.1)
Abdominal symptoms:	****	****
Vomiting, nausea, abdominal pain	2	0.3 (0.0 to 0.9)
Loose stools	1	0.1 (0.0 to 0.7)
Ear, nose, and throat: mild nose bleed	6	0.8 (0.3 to 1.7)
Ocular: itch, redness	1	0.1 (0.0 to 0.7)
Neurological: any	0	0.0 (0.0 to 0.5)
Cardiovascular: any	0	0.0 (0.0 to 0.5)

Given the concern about wheeze after vaccination with LAIV, we analysed the change in asthma control test score for the four weeks after vaccination, from baseline. The score was determined at both time points for 394/445 (89%) participants with a history of asthma or recurrent wheeze. The asthma control test score did not significantly change for those aged 12 years and over (median change 0, P=0.12, fig 2). In those aged 2-11 years, there was a small but significant improvement in the score after vaccination (median change 1, P<0.001). A similar improvement was also noted when the analysis was restricted to children under 5 years (median change 1, P<0.001).

**Figure fig2:**
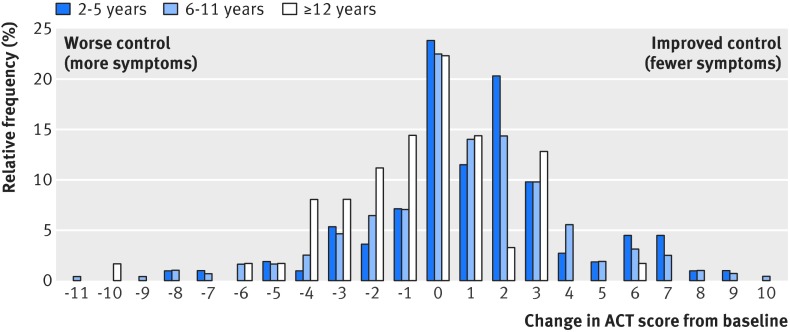
**Fig 2** Change in asthma control test (ACT) score at four weeks after immunisation with LAIV compared with baseline in young people with a history of asthma or recurrent wheeze

In the 29 participants who received a second dose of LAIV and who had complete follow-up, four experienced an adverse event within 72 hours after immunisation. Two participants experienced a flare in eczema; in one this also occurred after the first dose of LAIV.

## Discussion

### Systemic allergic reactions

In young people with egg allergy we did not observe any systemic allergic reactions or anaphylaxis after immunisation with quadrivalent LAIV. Anaphylaxis is defined as a “severe, life-threatening generalized or systemic hypersensitivity reaction.”^24^ ^25^ Thirty five percent of study participants had a history of anaphylaxis to egg: 20% had experienced respiratory and/or cardiovascular involvement. In this study, participants with previous anaphylaxis to egg were not found to be at a higher risk of AEFI (of allergic cause, or otherwise) with LAIV.

Together with previous studies,^26^ ^27^ the literature now reports 955 young people with egg allergy (including 338 (35%) with previous anaphylaxis to egg) who have received at least one dose of LAIV without an acute systemic reaction. This gives an upper 95% confidence interval for the incidence of acute systemic allergic reaction in young people with egg allergy in the general population of 0.39%, or less than 1 in 256 vaccinated young people with egg allergy. In young people with previous anaphylaxis to egg, the upper 95% confidence interval for the incidence of acute systemic reaction is 1.09%. The incidence of possible local, IgE mediated reactions is higher (1.2%) than that previously reported for people without egg allergy.^15^ However, these reactions were all mild, localised, and self limiting. Anaphylaxis to LAIV has been reported in adults (at a rate of 0.3 reactions per 100 000 doses), but none were related to egg allergy.^28^ We have previously reported that LAIV is unlikely to contain enough egg protein to trigger an IgE mediated allergic reaction in people with egg allergy.^29^ The risk of causing a systemic allergic reaction with quadrivalent LAIV therefore seems to be no greater in young people with egg allergy (including those with a history of anaphylaxis to egg) compared with those without egg allergy.

Following discussions with our local patient and public involvement panel, we chose to use an open design for this phase IV study, to maximise recruitment to the study. Input from the panel indicated that it would not be as acceptable to include a placebo arm, nor would many parents consent for their offspring to be immunised with inactivated influenza vaccine when a non-injectable alternative was available. Thus, we can only compare risk of adverse events with historical data.

This study confirms our previous findings that LAIV is unlikely to trigger a systemic allergic reaction in young people with egg allergy, with several important additions. Our earlier study provided initial data relating to the safety of LAIV in 282 young people with egg allergy^26^; however, the trivalent vaccine used in that study did not have detectable egg protein, thus the safety profile may have been due to a lack of egg protein in the vaccine batches used. In this study, the majority of the LAIV batches contained detectable ovalbumin. This, combined with the larger cohort size and a more representative population of young people with egg allergy, achieved by recruiting from non-tertiary allergy clinics, provides a stronger evidence base to support the safety of using LAIV in young people with egg allergy. In theory it is possible that previous vaccination with LAIV might result in sensitisation and an increased risk of subsequent reaction in future years. In this study, 24% of the cohort received LAIV in 2013/14, and this was not associated with an increased risk of adverse events. Reassuringly, the rate of delayed adverse events in this study is similar to that previously reported after LAIV in non-atopic young people (table 2).^4^ ^5^ ^6^ ^7^ ^9^ ^15^ ^28^

**Table 2 tbl2:** Rates of adverse events within 72 hours after immunisation using LAIV compared with rates in the literature

Symptoms within 72 hours	No (%) in study (n=779)	Rates in literature (%)
Allergic reaction (mild symptoms) only	9 (1.2)	0.02
Allergic reaction: anaphylaxis	0 (0)	0
Fever	39 (5.0)	5.4
Nasal symptoms	141 (18.1)	31
Wheeze (parent reported)	29 (3.7)	NR
Wheeze requiring treatment by doctor	3 (0.4)	0.2
Lower respiratory tract symptoms	62 (8.0)	NR
Eczema flare	22 (2.8)	NR

NR=not reported.

Rates reported as proportion of total number of doses given, to be consistent with method of reporting used in existing literature.^15^

### Wheeze after LAIV

Guidelines from North America currently recommend against the use of LAIV in children under 5 years with a history of an episode of wheezing in the previous 12 months,^16^ owing to concerns that the vaccine might cause wheezing in susceptible children, something not consistent with published data.^4^ ^5^ ^6^ ^15^ ^30^ ^31^ An analysis of two randomised, multinational trials, in 1940 children aged 2 to 5 years with asthma or a history of wheezing, found no difference in the incidence of wheezing after vaccination between those who received LAIV and those who received trivalent influenza vaccine.^32^ However, both trials excluded children with wheeze in the 42 days before receiving LAIV. Furthermore, previous studies have used “medically significant wheeze” in the 42 days after vaccination as the outcome measure for lower respiratory tract symptoms. Although this may be a measure of more concerning wheeze, it is insensitive, as many parents of children with recurrent wheezing will manage their child’s symptoms at home without recourse to a medical professional. Parent reported wheeze is common in the autumn and winter months (when immunisation with LAIV is indicated). In this study, we only excluded children with acute wheezing in the previous three days, a more feasible scenario in terms of a targeted immunisation campaign. We did observe a higher rate of parent reported lower respiratory tract symptoms in children aged 2 to 5 years, but this did not reach statistical significance. To explore this further, we used the asthma control test questionnaire to assess asthma symptoms, including wheeze, in the four weeks before and after vaccination . We did not observe a significant increase in lower respiratory tract symptoms in children under 5 years of age receiving LAIV, nor was there a worsening in asthma control test scores. These data suggest that LAIV is safe in children with a history of asthma or recurrent wheeze in whom symptoms are well controlled.

### Strengths and limitations of this study

Our study population was recruited from a large number of secondary and tertiary allergy centres in the UK, and may therefore represent a cohort of young people with more severe allergy requiring specialist input. We therefore expect our findings to be applicable to a wider population of young people with egg allergy, including those with more mild allergy managed in primary care. We excluded those who had previously required ventilation on intensive care after an anaphylaxis triggered by egg. This, however, is an atypical occurrence, and we excluded no child because of this criterion. Anaphylaxis to food is not uncommon, with an estimated incidence in children with food related allergy of 0.20 (95% confidence interval 0.09 to 0.43) cases per 100 person years.^33^ In contrast, fatal anaphylaxis is a rare event (although unpredictable), with an estimated incidence of 1.81 (95% confidence interval 0.94 to 3.45) cases per million person years.^34^ There are approximately 10 fatalities due to food anaphylaxis in the UK per annum,^35^ compared with an annual average of 30-40 admissions to intensive care due to food anaphylaxis (data obtained from UK Health and Social Care Information Centre).^36^ The published data indicate that children with egg allergy with a history of anaphylaxis are not more sensitive to lower doses of egg than those with only previous mild reactions.^37^ Taken together, these data suggest that LAIV is likely to be well tolerated even in those few children with previous anaphylaxis to egg requiring intensive care, although arguably it is reasonable to expect such children to be vaccinated within an appropriate healthcare facility.

### Conclusions and policy implications

This study provides evidence to support the revised Department of Health guidance for the 2015/16 season that, with the exception of children “with severe anaphylaxis to egg which has previously required intensive care, children with an egg allergy can be safely vaccinated with Fluenz Tetra® in any setting (including primary care and schools).”^12^ As with all settings providing vaccination, facilities should be available and staff trained to recognise and treat anaphylaxis. Furthermore, the vaccine is appropriate for use in children at risk of wheeze, in whom symptoms are well controlled and with no evidence of active wheezing in the 72 hours before immunisation.

**What is already known on this topic**

Egg allergy is common, affecting 2-6% of preschool children
An intranasal vaccine (live attenuated influenza vaccine, LAIV) has been introduced into the UK paediatric vaccination schedule, but there are limited safety data for its use in young people with egg allergy and/or asthma
Some guidelines recommend against using LAIV in children under 5 years with a history of recurrent wheeze or asthma

**What this study adds**

LAIV did not cause any systemic allergic reactions in this cohort of young people with egg allergy
LAIV seems to be well tolerated in young people with a diagnosis of asthma or recurrent wheeze, provided that lower respiratory symptoms are well controlled

## Notes

We thank our data monitoring committee (Glenis Scadding (chair), Andrew Riordan, Giuseppina Rotiroti, and Andre Charlett) and our trial steering committee (Nicola Brathwaite (chair), Diab Haddad, and Hazel Gowland). We also thank our co-investigators in the SNIFFLE-2 Study team and the UK Paediatric Vaccine Group for their support, as well as Public Health England colleagues for their support in data management: Samuel Lattimore, Deborah Cohen, Rashmi Malkani, and Teresa Gibbs. Finally, we thank the many parents and young people who participated in the study.

This report follows the TREND guidelines for the reporting quality of non-randomised evaluations of a public health intervention. Des Jarlais DC, Lyles C, Crepaz N, and the TREND Group (2004). Improving the reporting quality of nonrandomized evaluations of behavioral and public health interventions: the TREND statement. *Am J Public Health* 2004;94:361-6.

The SNIFFLE-2 Study Investigators are: Richard Briggs (Warrington & Halton Hospitals NHS Foundation Trust), John Chapman (James Paget University Hospitals NHS Foundation Trust), Rahul Chodhari (Royal Free Hospital NHS Foundation Trust), Jonathan Cohen (University College London Hospitals NHS Foundation Trust), Iain Darwood (Wye Valley NHS Trust), Brenda DeWitt (Leeds Teaching Hospitals NHS Trust: Leeds Children’s Hospital), Christine Doyle (Alder Hey Children’s NHS Foundation Trust), Michel Erlewyn-Lajeunesse (University Hospital Southampton NHS Foundation Trust), Roisin Fitzsimons (NIHR Biomedical Research Centre at Guy’s and St Thomas’ NHS Foundation Trust), Keith Foote (Hampshire Hospital NHS Foundation Trust), James Gardner (Royal Free Hospital NHS Foundation Trust), Paul T Heath (Institute of Infection and Immunity, St George’s University of London), Donald Hodge (Leeds Teaching Hospitals NHS Trust: Leeds Children’s Hospital), Stephen M Hughes (Central Manchester University Hospitals NHS Foundation Trust), Nicola Jay (Sheffield Children’s Hospital NHS Foundation Trust), Susan Leech (King’s College Hospital NHS Foundation Trust), Colin Lumsden (Lancashire Teaching Hospitals NHS Foundation Trust), Nick Makwana (Sandwell and West Birmingham Hospitals NHS Trust), Louise J Michaelis (Great North Children’s Hospital, Newcastle upon Tyne Hospitals NHS Foundation Trust), Eleanor Minshall (Cambridge University Hospitals NHS Foundation Trust), Anita Modi (Luton and Dunstable University Hospital NHS Foundation Trust), Lee Noimark (Barts Health NHS Trust), Bernadette O’Connor (Ulster Hospital, South Eastern Health and Social Care Trust), KP Ramesh (Cambridgeshire Community Services NHS Trust), Martyn Rees (Shrewsbury and Telford Hospital NHS Trust), Jürgen Schwarze (University of Edinburgh and NHS Lothian), Matthew D Snape (NIHR Oxford Biomedical Research Centre and Oxford University Hospitals NHS Trust), Gary Stiefel (University Hospitals of Leicester NHS Trust), Huw M Thomas (University Hospitals Bristol NHS Foundation Trust), Paul J Turner (NIHR/Imperial Biomedical Research Centre and Asthma UK Centre in Allergic Mechanisms of Asthma, Imperial College London), Lynette Williams (Shrewsbury and Telford Hospital NHS Trust), and Natasha Zurick (Royal United Hospital Bath NHS Trust).

Ethical approval: This study was approved by the West Midlands-Edgbaston research ethics committee (14/WM/0159) and the parent/guardian of each participant gave written informed consent.

Data sharing: no additional data available.

Transparency: The manuscript’s guarantor (PJT) affirms that this manuscript is an honest, accurate, and transparent account of the study being reported; that no important aspects of the study have been omitted; and that any discrepancies from the study as planned (and, if relevant, registered) have been explained.

## Notes

Cite this as: *BMJ* 2015;351:h6291
