# Supplementary material for: Safety of live attenuated influenza vaccine in young people with egg allergy: multicentre prospective cohort study
Source: BMJ. 2015 Dec 8;351:h6291. doi: 10.1136/bmj.h6291 (PMC4673102; doi:10.1136/bmj.h6291)
Supplement: Supplementary file 1 [file turp029119.ww1_default.pdf]

Table E1: AEFI reported within 2 hours of LAIV administration

Study ID number	Time to symptoms (minutes)	Description of events	IDMC review: possible allergic aetiology?	Causality
**BR/005**	20-60	Generalised urticarial following LAIV. Negative allergy testing and tolerated 2nd dose of LAIV. Reaction attributed to milk present in ingredients of snack eaten following 1st dose of LAIV (child has milk allergy).	NO	
**CA/011**	5-20	Red itchy nose	YES	Probable
**ED/054**	5-20	Itchy naso-oropharynx. Facial flushing; no hives	YES	Probable
**LE/005**	5-20	Felt as if wanted to sneeze, but no actual sneezing. No significant change in TNSS.	NO	
**LE/020**	5-20	Pre-existing nasal symptoms due to mild cold.	NO	
**LP/001**	5-20	Large hive on face-disappeared in minutes. Scratching both sides of face, transient	YES	Possible
**LP/016**	20-60	Itchy erythema/ hives on forehead; eczema flare on arms 30 mins post LAIV	YES	Probable
**NW/018**	20-60	Local rhinitis symptoms at 30 mins post LAIV	YES	Possible
**OX/006**	1-5	Itchy nose and skin - ?due to contact reaction, no visible urticaria	YES	Possible
**OX/020**	20-60	Single episode of vomiting, 45 mins after LAIV. Does not meet BCC criteria for systemic reaction.	NO	
**OX/022**	5-20	Contact urticarial reaction	YES	Probable
**RL/031**	5-20	Mother reported mild R facial swelling. Negative allergy testing and tolerated 2nd dose of LAIV without any similar symptoms.	NO	
**SF/025**	20-60	3 hives on chin 25 mins post LAIV, resolved in <15mins	YES	Probable
**UC/006**	5-20	Mild nasal itch, no change in TNSS. No objective symptoms. Tolerated 2nd dose without issue.	NO	
**UC/001**	60-120	Itchy tongue and neck	YES	Possible
**SM/066**	20-60	Sore 'tummy' 40mins post LAIV, onset shortly after eating a take-away from local restaurant. Family member (not receiving LAIV) had same symptoms.	NO	
**MA/023**	60-120	Not Itchy	NO	

Table E2: Incidence of AEFI of allergic aetiology, by factor of interest.

Factor	Level	n/N	P value
Age group (years)	2-4	5/360 (1.4%)	0.24
	5-11	2/334 (0.6%)	
	12-18	2/85 (2.4%)	
Physician-assessed clinical reaction to egg in previous 12 months	No	4/464 (0.9%)	0.50
	Yes	5/315 (1.6%)	
95% PPV criteria met for egg allergy within previous 12months	No	3/326 (0.9%)	0.74
	Yes	6/453 (1.3%)	
Previous anaphylaxis to egg	No	7/622 (1.1%)	1.00
	Yes	2/157 (1.3%)	
Previous reported reaction to aerosolised egg	No	6/709 (0.8%)	0.04
	Yes	3/70 (4.3%)	
Tolerance to baked egg in diet	No	5/436 (1.1%)	1.00
	Yes	4/343 (1.2%)	
Previous influenza vaccine (any)	No	3/410 (0.7%)	0.32
	Yes	6/369 (1.6%)	
Previous LAIV	No	7/591 (1.2%)	1.00
	Yes	2/188 (1.1%)	
Diagnosis of asthma or recurrent wheeze	No	3/334 (0.9%)	0.74
	Yes	6/445 (1.3%)	
Batch ovalbumin content >1.0 ng/ml	No	2/268 (0.7%)	0.73
	Yes	7/511 (1.4%)	
Batch ovalbumin content >0.3 ng/ml	No	1/112 (0.9%)	1.00
	Yes	8/667 (1.2%)	

Table E3: Incidence of delayed events within 72 hours of LAIV administration, by factor of interest.

Factor	Level	n/N	P
Age group (years)	2-4	112/360 (0.3%)	0.07
	5-11	93/334 (0.3%)	
	12-18	16/85 (18.8%)	
Physician-assessed clinical reaction to egg in previous 12 months	No	140/464 (30.2%)	0.20
	Yes	81/315 (25.7%)	
95% PPV criteria met for egg allergy within previous 12months	No	101/326 (31.0%)	0.17
	Yes	120/453 (26.5%)	
Previous anaphylaxis to egg	No	180/622 (28.9%)	0.55
	Yes	41/157 (26.1%)	
Previous reported reaction to aerosolised egg	No	199/709 (28.1%)	0.58
	Yes	22/70 (31.4%)	
Tolerance to baked egg in diet	No	120/436 (27.5%)	0.58
	Yes	101/343 (29.4%)	
Previous influenza vaccine (any)	No	123/410 (30.0%)	0.30
	Yes	98/369 (26.6%)	
Previous LAIV	No	176/591 (29.8%)	0.14
	Yes	45/188 (23.9%)	
Diagnosis of asthma or recurrent wheeze	No	92/334 (27.5%)	0.69
	Yes	129/445 (29.0%)	
Batch ovalbumin content >1.0 ng/ml	No	80/268 (29.9%)	0.51
	Yes	141/511 (27.6%)	
Batch ovalbumin content >0.3 ng/ml	No	28/112 (25.0%)	0.43
	Yes	193/667 (28.9%)	
